# Life Course Assessment of Area-Based Social Disadvantage: A Systematic Review

**DOI:** 10.3390/ijerph20216982

**Published:** 2023-10-27

**Authors:** Sarah A. Keller, Sarah Lim, William R. Buckingham, Amy J. H. Kind

**Affiliations:** 1 Center for Health Disparities Research, School of Medicine and Public Health, University of Wisconsin, Madison, WI 53726, USA; 2 Department of Population Health Sciences, School of Medicine and Public Health, University of Wisconsin, Madison, WI 53726, USA; 3 Wisconsin Alzheimer’s Disease Research Center, School of Medicine and Public Health, University of Wisconsin, Madison, WI 53726, USA; 4 Department of Medicine, Geriatrics Division, School of Medicine and Public Health, University of Wisconsin, Madison, WI 53726, USA

**Keywords:** social disadvantage, neighborhood disadvantage, area disadvantage, life course, longitudinal studies

## Abstract

Area-based social disadvantage, which measures the income, employment, and housing quality in one’s community, can impact an individual’s health above person-level factors. A life course approach examines how exposure to disadvantage can affect health in later life. This systematic review aimed to summarize the approaches used to assess exposure to area-based disadvantage over a life course, specifically those that define the length and timing of exposure. We reviewed the abstracts of 831 articles based on the following criteria: (1) whether the abstract described original research; (2) whether the study was longitudinal; (3) whether area-based social disadvantage was an exposure variable; (4) whether area-based social disadvantage was assessed at multiple points; and (5) whether exposure was assessed from childhood to older adulthood. Zero articles met all the above criteria, so we relaxed the fifth criterion in a secondary review. Six studies met our secondary criteria and were eligible for data extraction. The included studies followed subjects from childhood into adulthood, but none assessed disadvantages in late life. The approaches used to assess exposure included creating a cumulative disadvantage score, conducting a comparison between life course periods, and modeling the trajectory of disadvantage over time. Additional research was needed to validate the methodologies described here, specifically in terms of measuring the impact of area-based social disadvantage on health.

## 1. Introduction

It is well documented that the burden of adverse health conditions is overrepresented in populations adversely impacted by historical and modern inequities. For example, cardiovascular disease [1], diabetes [2], and rates of COVID-19 infection [3] have all been linked to social inequality. These differences in health are thought to result from a combination of multi-level factors at both the individual and area levels [4]. Area-based social disadvantage, which measures the income, education, employment, and housing quality of one’s neighborhood or community, can impact an individual’s health over and above individual-level social factors [5]. A life course approach can examine how these physical and social exposures, which may accumulate over time and cluster around significant periods like early childhood, impact health and disease risk in later life [6,7].

Our goal for this review is to understand how, if at all, life course theories have been applied to the assessment of area-based social disadvantage. We discuss the approaches used in the literature to assess an individual’s exposure to area-based disadvantage over a life course, with a particular focus on how the length and timing of exposure are defined. We extracted the data sources used to create measures of area-based social disadvantage and the individual components of these metrics. We also examined the specific methods used to summarize life course exposure in the study populations, and we discussed the benefits and limitations of each. Based on existing life course theory [6,7], we expect these approaches to fall into two categories: the creation of a cumulative or average disadvantage score or a comparison of the measures of disadvantage between specific life course periods. This field of study is still novel, so we hope that a thorough accounting of the literature in this area will help investigators choose the methodology that is most appropriate for their work, as well as help in ultimately advancing the research forward.

## 2. Materials and Methods

We performed a systematic review of methodologies that have been used in the assessment of life course area-based disadvantage, specifically those quantifying the length and timing of exposure. We asked the following research question: Which methodological approaches have been used to assess an individual’s exposure to area-based disadvantage over a life course? We were particularly interested in the way prior work measured cumulative life course exposure to area-based disadvantage, as well as the timing of that exposure.

### 2.1. Search Strategy

A preliminary search in PubMed (Medline) was used to identify relevant search terms and Medical Subject Heading (MeSH) terms to capture the concepts related to area-based social disadvantage and life course assessment. These included “neighborhood disadvantage”, “life course perspective”, and “multiple deprivation”. We also used search terms to exclude surveys, questionnaires, and interviews to ensure the returned records contained objective, area-based measures of disadvantage instead of subjective self-report measures. A search strategy was finalized in PubMed on 8 April 2022. The search terms and syntax were adapted and used in searches on 9 April 2022 for the following additional databases: Scopus, PsychINFO, and the Sociology Research Database (SocINDEX). The final search strategy used is available in Appendix A. This review followed the guidelines established by PRISMA [8]. Our review did not meet the inclusion criteria for registration in the International Prospective Register of Systematic Reviews (PROSPERO) [9] because our review synthesizes methods not directed toward a specific intervention or outcome. Therefore, our study was not registered, and the protocol was not published.

### 2.2. Study Selection

The citations returned from the database searches were imported into citation management software, and duplicate records were removed. The remaining records were exported into Brown University’s Abstrackr web application, which manages records for the title and abstract screening [10]. Two reviewers independently reviewed 100% of the titles and abstracts for adherence to the inclusion criteria to reduce opportunities for bias [11]. 

### 2.3. Eligibility Criteria

The title and abstract screen evaluated articles based on the following criteria: (1) if the abstract described original research; (2) if that research was longitudinal in nature; (3) if an area-based metric of social disadvantage was treated as an exposure variable; (4) if the area-based metric of social disadvantage was assessed at multiple time points during the study; and (5) if that metric was assessed over an entire life course. For the purposes of our review, an area-based measure of social disadvantage was defined as a metric when it used small geographic units to detect nuanced differences in income, education, living conditions, and/or social support in a population [12].

Life course assessment was defined as at least one measurement of area-based social disadvantage during childhood (<18 years old), one during adulthood (18–65 years), and one during older adulthood (>65 years) in our initial review. However, we performed a second exploratory review using the same records identified by our search strategy and expanding the life course assessment criterion. Rather than requiring a measurement of area-based social disadvantage in childhood, adulthood, and older adulthood, abstracts in our second review were eligible if a measurement was made in at least two of these three life course periods. Results from both our primary and secondary reviews are presented below.

## 3. Results

### 3.1. Article Selection

**Figure 1:**
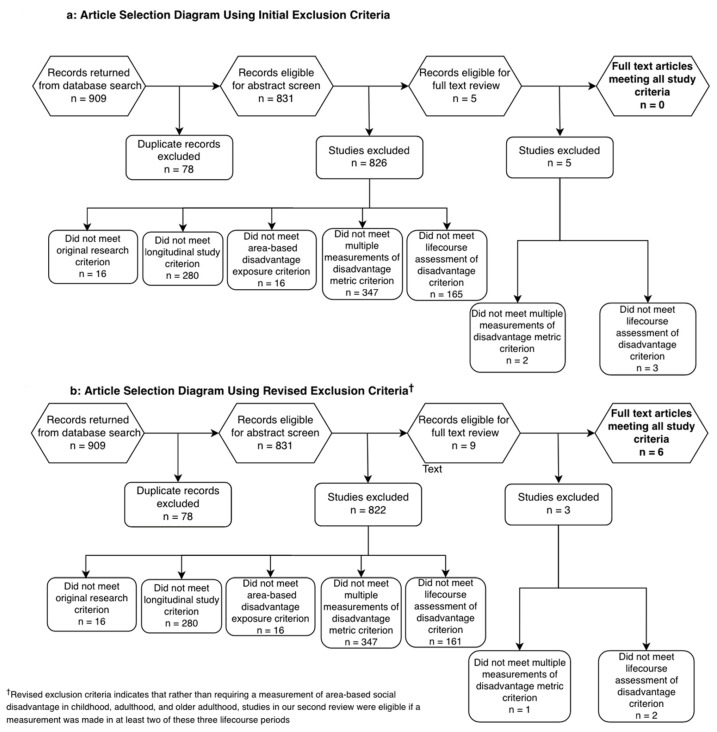
Article selection diagram.

The article selection process for this review is presented in Figure 1. Our database search identified 909 records, 831 eligible for title and abstract screening after duplicate removal. Five articles appeared to meet our five inclusion criteria and were deemed eligible for full-text review. Upon review of the full manuscripts of each article, two were excluded for not meeting the multiple measurements of the disadvantage metric criterion, and three were excluded for not meeting the life course assessment of the disadvantage criterion. Zero articles met our complete criteria to be eligible for data extraction. However, in service of our aim for this review, which is to act as a guide for future investigators, we determined it may be most useful to perform a second review with slightly relaxed criteria.

In this secondary review, we revised our fifth, most strict criterion such that a study is now eligible if a measurement of an area-based disadvantage metric is taken in two of the following three life course periods: childhood, adulthood, and older adulthood. A re-screen of titles and abstracts with this new criterion returned nine articles eligible for full-text review. Three articles were excluded at this stage—one for not meeting the multiple measurements of the disadvantage metric criterion and two for not meeting the revised life course assessment of the disadvantage criterion. Six articles met our complete criteria and were eligible for data extraction [13,14,15,16,17,18].

### 3.2. Data Extraction

Table 1 presents a summary of the study characteristics in the eligible articles. The included studies were published between 2013 and 2021. Five of the six studies were located in Europe—two in the United Kingdom [16,18], one in Sweden [14], one in Finland [15], and one in the Netherlands [17]—and one was based in the United States [13]. All six studies utilized data from prospective cohort studies.

All six studies characterized their metric of an area-based disadvantage as “neighborhood disadvantage”, a small unit of geography that provides more granular information and potentially more nuance than larger units such as cities or counties [12]. Two studies used previously existing metrics of the area-based disadvantage: the Townsend Deprivation Index [16,19] and the Indices of Multiple Deprivation [18,20]. The remaining four articles described the construction of new metrics for their studies using publicly available statistics and geographic measures [13,14,15]. The length of follow-up of the cohorts in the included studies ranged from 5 to 42 years, with an average length of follow-up of 24.3 years. Five of the six studies followed participants from childhood to adulthood [14,15,16,17,18], where adulthood is defined as between the ages of 18 and 64, and one study followed participants from adulthood into older adulthood [13], age 65+.

Outcomes measured varied considerably across studies. Four studies used biological and clinical measures like body mass index (BMI) [16], functional independence and decline [13], diabetes onset [15], and allostatic load [14]. Others used measures related to area-based social disadvantage, including work commitment and unemployment [17] and perceived neighborhood disadvantage [18].

Most included studies used a composite index integrating multiple components to create their metric of disadvantage. Table 2 evaluates these metrics by comparing how their components fall across seven domains of area-based disadvantage identified by Buckingham et al. (2021): income, education, housing, employment, neighborhood structure, demographic makeup, and health. Metrics of the included studies had components covering an average of four domains, ranging from one to six domains. Employment was the domain most commonly included, used by five of the six metrics [13,14,15,16,18], followed by education [13,14,15,18], housing [15,16,17,18], and neighborhood structure [13,14,16,18], used by four metrics each. One study used a single measure—average neighborhood property value—which falls in the housing domain [17].

The approach used to measure the length and timing of exposure to area-based social disadvantage in the included studies is presented in Table 3. Three studies evaluated the level of disadvantage study participants were exposed to through a cumulative approach, in that they summed the measurements of the metric in question at each time point for which they were available [13,14,17]. Two of these three calculated a mean from each measurement of the disadvantage variable [13,14], while one study counted the number of months spent over a certain threshold and quantified the length of exposure as a continuous variable ranging from no to constant exposure [17]. Two studies defined specific life course periods and examined the impact of the timing of exposure on their outcome, in addition to a cumulative average across the entire follow-up period [15,16]. One study looked at the trajectory of measured exposure to neighborhood-level disadvantage and their participants’ perceived disadvantage over the observation period using trajectory modeling methods [18].

## 4. Discussion

This review synthesized methodologies used across the published literature to assess exposure to life course area-based social disadvantage. There is increasing emphasis in health research on understanding the impact of area-based social disadvantage on various health outcomes, but there is no consensus on how to measure the length and timing of exposures across the life course. We identified six studies that capture a metric of area-based disadvantage over the life course to understand the current methodological approaches used by investigators and gaps to be targeted in future research.

No studies met the initial array of inclusion criteria, largely due to a lack of measures across the full life course. Because of this, none of the included studies follow participants from childhood to beyond the age of 65. This may exclude life course periods important to one’s exposure to social disadvantage, such as aging out of the workforce or end-of-life care. This surprising gap could be indicative of some of the inherent challenges of life course epidemiological studies. These include a lack of access to cohorts with repeated measures of the appropriate exposures and outcomes, limited data outside of specific time points such as early and late life, and the complex analytical considerations that must be made for these data [6]. The lack of results in our initial search points towards a significant gap in the literature for studies that measure area-based disadvantage across the entire life course.

A secondary search leveraging a relaxed life course criterion returned six studies. These studies were all published between 2013 and 2021, indicating that life course assessment of disadvantage is of growing interest. All but one of the studies used data from European countries, which frequently use area-based metrics of disadvantage to understand disparities and deliver services nationwide. This is in contrast to the United States, where no such metric is utilized by the federal government [12]. The variation in outcomes measured in the included studies demonstrates the broad applicability of area-based social disadvantage across disciplines and research interests.

Three of the six studies created their own metric of area-based disadvantage for the purposes of their research [13,14,15,17], while three utilized existing metrics [15,16,18]. These three metrics, the Townsend Deprivation Index [19], the Indices of Multiple Deprivation [20], and Statistics Finland [21], are all frequently used by government entities in their respective countries. These have the benefit of being well validated within their national population to describe the concentration of disadvantage in small geographic units across a range of domains over time (Table 2). Alternatively, it may be necessary for researchers to construct their own metrics when such metrics do not exist at the national level, do not capture the necessary domains to answer the research question at hand, or are not able to capture social disadvantage over time. It should be noted that these metrics may be highly specialized for a particular geographic region or population and may require additional validation before being used outside of the contexts for which they were created.

The cumulative approach allows researchers to sum a lifetime of exposure into a single number, allowing for simple comparison across participants. However, our included studies demonstrate that there are several methods to accomplish this, including mean or median level of exposure and time spent at or above a particular threshold of disadvantage. Additional research could validate these or other approaches in life course assessment of area-based social disadvantage and help researchers determine which methods are most appropriate for their studies.

Making comparisons within and between different life periods allows researchers to examine the impact of sensitive or critical periods on their outcome of interest or isolate periods in which there are large fluctuations in disadvantage, such as the transition from childhood to young adulthood. Cutoff points for these life course windows should also be carefully selected, perhaps with respect to developmental or social milestones such as the development of gross motor skills in young children or retirement age for older adults. Further research could guide investigators in choosing cutoff points when these milestones are not relevant to their research question or study population.

Our findings are consistent with other reviews examining the impact of area-based disadvantage on various outcomes. A recent scoping review of the effect of social disadvantage on neuropathologically confirmed cerebrovascular disease found a lack of longitudinal study designs and inconsistent methodologies overall [22]. A European review of the association between area-level disadvantage and suicidal behaviors found only one longitudinal study that met their inclusion criteria [23]. A review of how area-level socioeconomic status impacts substance use outcomes also returned primarily cross-sectional designs [24]. Notably, none of the included studies in the above reviews would meet either the primary or secondary criteria of the present review.

Our work adds to the growing number of researchers calling for more longitudinal studies of area-based social disadvantage using clearly justified methodologies, which requires collaboration across sectors and disciplines. Government bodies can invest in indices of disadvantage and assist with the regular collection of these measures while making data available for health research. Investigators creating longitudinal cohort studies can ensure that metrics of area-based disadvantage are included in follow-up visits. Geographers, biostatisticians, and epidemiologists can assist with the creation of area-based disadvantage indices and guide the assessment of life course exposure. Using the momentum of interest in this area, we can create standard methodologies to promote rigorous research.

### Limitations

While our review is the first of its kind to the best of our knowledge, this work also has its limitations. First, our review searched only academic health databases, meaning that it is possible that there are studies present in databases for other disciplines or white and gray literature that would meet our criteria. There may also be relevant articles published in a language other than English that we could not assess.

## 5. Conclusions

In this literature review, it was notable that no studies included a comprehensive life course assessment of area-based social disadvantage. While no studies met our initial criteria, the six that met the criteria of the secondary search provided some guidance on life course assessment of area-based social disadvantage and its impact on health outcomes. Critically, all six studies found an association between their metrics and outcomes of interest, despite variation in the methodologies and metrics used, indicating the relevance of this work. Continued investment to fund the measurement of area-based disadvantage in prospective cohort studies is crucial to maintaining representative samples of participants that can be followed over the life course.

Future studies could address gaps in the literature by utilizing an area-based metric of disadvantage tailored to the United States or regions outside of Europe. While longitudinal studies, in general, are greatly needed, studies that attempt to capture the disadvantage across the entire life course, from childhood to older adulthood, would be particularly useful. While following a cohort into old age may present methodological challenges, this life course period may be especially sensitive to changes in area-based disadvantage and ultimately impact health at the end of life.

We found that there does not seem to be a consensus in the literature on how to assess lifetime exposure to disadvantages. Future work can focus on validating these and novel methodologies to evaluate exposure to disadvantages over the life course, creating gold-standard mathematical approaches to further the science in this area. Such work is necessary to understand the impact of area-based social disadvantage on health and to address existing disparities through policy.

## Figures and Tables

**Table 1:** Summary characteristics of included studies.

Study	Study Location	Metric of Area-Based Deprivation	Metric Data Source	Metric Components	Main Findings
*Cumulative Exposure to Neighborhood Context: Consequences for Health Transitions Over the Adult Life Course* Clarke et al. (2013) [13]	USA	n/a	U.S. Census Bureau	Poverty, unemployment, female-headed families, households receiving public assistance income, proportion of African Americans	Neighborhood disadvantage is associated with both functional decline and death, irrespective of the affluence and ethnic composition of the neighborhood
*Life-Course accumulation of neighborhood disadvantage and allostatic load: Empirical integration of three social determinants of health frameworks * Gustafsson et al. (2014) [14]	Lulea, Sweden	n/a	Small-Area Market Statistics (SAMS)	Income, housing allowance, wealth, unemployment, single-parent households, occupational status, and educational attainment	Cumulative neighborhood disadvantage over the life course was associated with allostatic load at age 43
*Social Determinants of Health and Cardiovascular Disease: Current State and Future Directions Towards Healthcare Equity * Kivimäki et al. (2018) [15]	Finland	n/a	Statistics Finland	Low education, unemployment rate, and proportion of people living in rented housing	Cumulative neighborhood disadvantage was associated with diabetes onset and its risk factors
*Life course neighborhood deprivation effects on body mass index: Quantifying the importance of selective migration * Murray et al. (2021) [16]	UK	Townsend Deprivation Index ^1^	UK censuses	Car access, home ownership, unemployment, and household overcrowding	Those living in more disadvantaged areas had higher BMIs at each subsequent follow-up period
*Neighbourhood poverty, work commitment and unemployment in early adulthood: A longitudinal study into the moderating effect of personality* Nieuwenhuis et al. (2016) [17]	Utrecht, The Netherlands	n/a	Statistics Netherlands	Average property values in a given neighborhood as a proxy for neighborhood poverty	The length of exposure to neighborhood disadvantage was associated with weaker commitment to work and higher unemployment
*How do perceived and objective measures of neighbourhood disadvantage vary over time? Results from a prospective-longitudinal study in the UK with implications for longitudinal research on neighbourhood effects on health * Yakubovich et al. (2020) [18]	County Avon, UK	Indices of Multiple Deprivation ^2^	UK National Statistics	Income Deprivation, Employment Deprivation, Education, Skills and Training Deprivation, Health Deprivation and Disability, Crime, Barriers to Housing and Services, Living Environment Deprivation	Both exposure to objective neighborhood disadvantage and participants’ perception of that disadvantage vary over time

^1^ Townsend P, Phillimore P, Beattie A. Health and Deprivation: Inequality and the North. Routledge; 1988 [19]; ^2^ Smith T, Noble M, Noble S, Wright G, McLennan D, Plunkett E. The English indices of deprivation 2015. London: Department for Communities and Local Government. Published online 2015:1-94 [20].

**Table 2:** Domains of area-based disadvantage metrics captured by included studies.

Domains	Metrics Utilizing Domains	Income	Education	Housing	Employment	Neighborhood Structure	Demographic Makeup	Health
	Clarke et al. (2013) [13]	X	X		X	X	X	
	Gustafsson et al. (2014) [14]	X	X		X	X		
	Kivimäki et al. (2018) [15]		X	X	X			
	Murray et al. (2021) [16]			X	X	X		
	Nieuwenhuis et al. (2016) [17]			X				
	Yakubovich et al. (2020) [18]	X	X	X	X	X		X

**Table 3:** Measurement of the length and timing of exposure to area-based social disadvantage in included studies.

Study	Total Length of Follow-Up Period	Frequency of Measurement of the Area-Based Social Disadvantage Variable	Assessment of Life Course Exposure
Clarke et al. (2013) [13]	15 years	Up to three measurements were taken during periods when participants transitioned between the four waves of the study	Created a cumulative average for the period between two studies waves, summing the neighborhood characteristics and dividing by the number of waves.
Gustafsson et al. (2014) [14]	27 years	Four measurements were collected when the participants were 16, 21, 30, and 43 years of age	Individual neighborhood indicators were Z transformed, and the mean of the indicator scores were taken at each measurement point. The four means were then Z transformed, and a mean cumulative neighborhood disadvantage score was calculated for the full study duration
Kivimäki et al. (2018) [15]	31 years	One measurement for each residential address available during the follow-up period	Time-weighted disadvantage Z scores were calculated from the residential data to measure exposure in childhood, adulthood, and cumulatively across the entire follow-up period
Murray et al. (2021) [16]	39 years	One measurement was collected at each follow-up point for the two cohort studies, up to six measurements. For cohort members of the National Child Development Study, follow-up occurred at age 11, 16, 23, 33, 42, and 5. For Members of the British Cohort Study, follow-up occurred at age 16, 26, 34, and 42.	Measurements were linked to Townsend Deprivation Index ^1^ scores at each age. Z scores were generated for each indicator and calculated their mean.
Nieuwenhuis et al. (2016) [17]	5 years	One measurement was taken at each residential address participants live in between ages 16 and 21	The number of months each individual spent in the 6-digit postcode areas with the lowest quintile of property value between ages 16 and 21 was calculated as a continuous variable ranging from 0 (no exposure) to one (constant exposure) during the period.
Yakubovich et al. (2020) [18]	18 years	Ten measurements were collected when the children of participating mothers were age 0, 1.75, 3, 5, 7, 10, 12, 14, 16.5, and 18	Researchers utilized the quintile ranks of the individual indicators and the total index score from the Indices of Multiple Deprivation ^2^ to compare participant neighborhoods relative to all other neighborhoods. A binary variable where 0 represents the three least deprived quintiles and 1 represents the two most deprived quintiles was generated for each of the 10 time points of measurement to model the latent trajectories of objective neighborhood deprivation over time

^1^ Townsend P, Phillimore P, Beattie A. Health and Deprivation: Inequality and the North. Routledge; 1988 [19]; ^2^ Smith T, Noble M, Noble S, Wright G, McLennan D, Plunkett E. The English indices of deprivation 2015. London: Department for Communities and Local Government. Published online 2015:1-94 [20].

## Data Availability

No new data were created or analyzed in this study. Data sharing is not applicable to this article.

## Appendix A. List of Search Terms

(((((((((((((((((((“neighbourhood disadvantage” [Title/Abstract])) OR (“neighborhood disadvantage” [Title/Abstract])) OR (“disadvantage index” [Title/Abstract])) OR (“spatial disadvantage” [Title/Abstract])) OR (“community disadvantage” [Title/Abstract])) OR (“locational disadvantage” [Title/Abstract])) OR (“geographic disadvantage” [Title/Abstract])) OR (remoteness [Title/Abstract])) OR (“concentrated poverty” [Title/Abstract])) OR (“area deprivation” [Title/Abstract])) OR (“area-based deprivation” [Title/Abstract])) OR (“area disadvantage” [Title/Abstract])) OR (“geographic socioeconomic disadvantage” [Title/Abstract])) OR (“Neighborhood-level socioeconomic disadvantage” [Title/Abstract])) OR (“multiple deprivation” [Title/Abstract]))))) AND (((“Longitudinal Studies” [Mesh])) OR “Life Course Perspective” [Mesh] OR “cohort study” [Title/Abstract] OR “longitudinal study” [Title/Abstract] OR “longitudinally” [Title/Abstract] OR “prospective study” [Title/Abstract] OR “retrospective study” [Title/Abstract] OR “lifecourse perspective” [Title/Abstract]) NOT ((“Surveys and Questionnaires” [Mesh]) OR “Interviews as Topic” [Mesh] OR “questionnaire” [title/abstract] OR interview [title/abstract]).
